# The Validation of the Nomophobia Questionnaire Using a Modern Psychometric Approach: An Item Response Theory Analysis of 5087 Participants

**DOI:** 10.1002/brb3.70622

**Published:** 2025-06-10

**Authors:** Haitham Jahrami, Khaled Trabelsi, Achraf Ammar, Nicola Luigi Bragazzi, Hadeel Ghazzawi, Waqar Husain, Maha M. AlRasheed, Seithikurippu R. Pandi‐Perumal, Amir Pakpour, Zahra Saif, Michael V. Vitiello

**Affiliations:** ^1^ Government Hospitals Manama Bahrain; ^2^ Department of Psychiatry, College of Medicine and Health Sciences Arabian Gulf University Manama Bahrain; ^3^ High Institute of Sport and Physical Education of Sfax University of Sfax Sfax Tunisia; ^4^ Department of Movement Sciences and Sports Training, School of Sport Science the University of Jordan Amman Jordan; ^5^ Department of Training and Movement Science, Institute of Sport Science Johannes Gutenberg‐University Mainz Mainz Germany; ^6^ Research Laboratory, Molecular Bases of Human Pathology, LR19ES13, Faculty of Medicine of Sfax University of Sfax Sfax Tunisia; ^7^ Human Nutrition Unit Department of Food and Drugs University of Parma Parma Italy; ^8^ Nutrition and Food Technology Department, School of Agriculture the University of Jordan Amman Jordan; ^9^ Department of Humanities, Islamabad Campus COMSATS University Islamabad Islamabad Pakistan; ^10^ Clinical Pharmacy Department, College of Pharmacy King Saud University Riyadh Saudi Arabia; ^11^ Centre for Research and Development Chandigarh University Mohali India; ^12^ Division of Research and Development Lovely Professional University Phagwara India; ^13^ Department of Nursing, School of Health and Welfare Jönköping University Jönköping Sweden; ^14^ Department of Psychiatry & Behavioral Sciences University of Washington Seattle Washington USA

**Keywords:** Item Response Theory (IRT), Nomophobia, Nomophobia Questionnaire (NMP‐Q), Psychometric Analysis, Reliability, Validity

## Abstract

Nomophobia, or the anxiety of being separated from one's mobile phone, is an emerging psychological condition in our digital age. The 20‐item Nomophobia Questionnaire (NMP‐Q) was developed to assess nomophobia symptoms. This study utilized item response theory (IRT) and classical test theory (CTT) methods to evaluate the NMP‐Q's psychometric properties. Data were collected from 5087 adults who completed the NMP‐Q. The CTT analyses included the computation of correlation coefficients, including McDonald's *ω*, Cronbach's *α*, Guttman's *λ*2, Guttman's *λ*6, greatest lower bound, average interitem correlation, Feldt–Gilmer coefficient, Feldt–Brennan coefficient, and Raju's *β*. IRT analyses included graded response modeling of items, test information function, differential item functioning, and reliability. CTT analysis revealed high reliability coefficients across nine metrics and a four‐factor model with good fit indices. IRT analysis showed a strong item fit to the polytomous graded model, indicating the questionnaire's robustness. Based on IRT (both uni‐ and multidimensional IRT) analyses, we propose a 10‐item short version (NMP‐Q Short), demonstrating high correlation (*r* = 0.97) with the original NMP‐Q. Our findings provide very good support for the NMP‐Q's reliability and construct validity. The items displayed good discrimination and difficulty parameters. The second 10 items of the NMP‐Q appeared to be the most informative. The proposed NMP‐Q Short offers a valuable option for efficient/rapid and accurate screening. However, additional validation and sensitivity testing are warranted to further establish its usefulness.

## Graphical abstract

Using item response theory, this study validates the Nomophobia Questionnaire's reliability in 5087 participants and proposes a 10‐item NMP‐Q Short (*r* = 0.97 with original NMP‐Q), offering an efficient tool for rapid nomophobia screening.

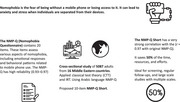

## 1 Introduction

Portable electronic mobile devices are no longer considered a luxury but rather a necessity in a modern 24‐h society (Aydin and Kuş 2023; Daraj et al. 2023). Excessive attachment to, and anxiety about being away from, mobile devices has been termed nomophobia (NMP), etymology: no‐mobile‐phone phobia (León‐Mejía et al. 2021). NMP refers to the fear or worry of being without access to one's mobile phone or being unable to use it (León‐Mejía et al. 2021). Individuals with high levels of NMP constantly check their phones, feel anxious when separated from their devices, and engage in excessive phone usage (Jahrami et al. 2021). Research suggests NMP is a growing problem, particularly among adolescents and young adults. A recent worldwide meta‐analysis of 52 original research studies involving ∼47.5 thousand participants revealed that 50% endorsed moderate and 20% severe symptoms of NMP (Jahrami et al. 2022).

The Nomophobia Questionnaire (NMP‐Q) has been the primary research measure used in research to assess symptoms of NMP (Yildirim and Correia 2015; Yildirim et al. 2016). The NMP‐Q was originally developed in English by Yildirim and Correia (2015) (Yildirim et al. 2016) and subsequently translated into Arabic (Al‐Balhan et al. 2018), German (Coenen and Görlich 2022), Greek (Gnardellis et al. 2023), Persian (Lin et al. 2018), Chinese (Gao et al. 2020), Portuguese (Galhardo et al. 2020; Gutiérrez‐Puertas et al. 2019), Italian (Bragazzi et al. 2016), Spanish (Gutiérrez‐Puertas et al. 2016), and Turkish (Yildirim et al. 2016). The NMP‐Q contains 20 items, each scored on a Likert scale from 1 (*strongly disagree*) to 7 (*strongly agree*); these scores are summed to obtain a total score (Yildirim and Correia 2015; Yildirim et al. 2016). The NMP‐Q has a maximum score of 140; the higher the score, the more severe the NMP. A score of 20 or less indicates no NMP, a score of 21–59 indicates mild NMP, a score of 60–99 indicates moderate NMP, and a score of 100 (or more) indicates severe NMP (Yildirim and Correia 2015; Yildirim et al. 2016).

The NMP‐Q has been shown to have strong reliability and validity using classical test theory (CTT) methods such as assessing internal consistency, test–retest reliability, and factor analysis (Yildirim and Correia 2015). According to a reliability generalization meta‐analysis, the NMP‐Q has excellent internal consistency, with a Cronbach's *α* value of 0.93 for the entire questionnaire (Jahrami et al. 2023). The same meta‐analysis showed that the subscales also demonstrated good reliability, with Cronbach's *α* values of 0.91 [0.88, 0.93] for Subscale 1, 0.84 [0.80, 0.88] for Subscale 2, 0.83 [0.78, 0.88] for Subscale 3, and 0.83 [0.80, 0.85] for Subscale 4 (Jahrami et al. 2023). The meta‐analysis was based on 13 studies with a combined sample size of 15,929 participants for the full scale and 10 studies with 12,840 participants for the subscales that included seven languages (Jahrami et al. 2023).

The factor structure of the NMP‐Q remained robust in all available language versions, with a satisfactory structure supporting its multidimensionality (Jahrami et al. 2023). Specifically, four factors causing anxiety when away from one's phone consistently emerge, including not being able to communicate (Factor 1), losing connectedness (Factor 2), not being able to access information (Factor 3), and giving up convenience (Factor 4) (Jahrami et al. 2023).

The use of modern approaches for validating the NMP‐Q is scarce; thus, very little is known about the performance of the individual items of the NMP‐Q. To illustrate, both Person A and Person B scored 77 on the NMP‐Q (i.e., severe levels of NMP). Person A scored 7 (*strongly agree*) in the first set of 10 items and 1 (*strongly disagree*) in the second set of 10 items. Person B scored 1 (*strongly disagree*) in the first set of 10 items and 7 (*strongly agree*) in the second set of ten items. It is misleading to propose that Person A and Person B have identical levels of NMP severity. Information about item differences, item discrimination, item importance, item difficulty, and other related item‐level aspects remains unexplored.

The purpose of this study is to use modern validation techniques of item response theory (IRT) to further advance our knowledge about NMP. IRT can identify poorly performing items that might be overlooked when focused on the full test. IRT can also evaluate the scale's precision at different levels of the concept being evaluated (Zanon et al. 2016). Even for a test with robust classical psychometrics, IRT can optimize the instrument, revealing the most useful measurement ranges and pinpointing areas that might be improved. In contrast to CCT approaches that rely on the observed total score, IRT takes a model‐based approach to provide item‐ and test‐level measurement diagnostics that classical methods alone do not provide (Zanon et al. 2016). Thus, IRT offers additional value by offering deeper insight into a test's strengths and weaknesses. Through item characteristic curves (ItCCs), researchers can visualize how each item performs across the entire ability spectrum, ensuring that the scale provides consistent measurement precision throughout the latent continuum (Zanon et al. 2016). IRT offers the advantage of parameter invariance, meaning that item parameters behave similarly across different populations after appropriate standardization, allowing for more valid cross‐group comparisons than possible with CTT approaches (Zanon et al. 2016). The test information function will help identify at which levels of the latent trait the NMP scale provides the most precise measurement, potentially revealing if the instrument is more effective at measuring certain ranges of the construct (Zanon et al. 2016). Thus, IRT offers additional value by providing deeper insight into a test's strengths and weaknesses at both the item and scale levels, ultimately leading to a more refined and precise measurement instrument.

This study addresses this gap by applying both CTT and IRT methodologies to rigorously evaluate the NMP‐Q's psychometric properties in a large sample of 5087 adults from 16 Middle Eastern countries, using a validated Arabic version of the questionnaire.

## 2 Methods

### 2.1 Study Design

This study utilized a cross‐sectional design using an online questionnaire, with data collection conducted during the second quarter of 2021 in 16 Middle Eastern countries.

### 2.2 Sample Selection and Participants

Multiple instant messaging channels and social media platforms were used to recruit participants using a convenience‐based snowball sample strategy. Data were collected from adult participants aged 18 and above from 16 Middle Eastern countries. The study sample included 5087 participants (57% females), with a mean age of 33 ± 12 years (range 18–72 years).

### 2.3 Instrumentation or Measures

We collected standard socio‐demographics, including country of origin, age, sex, marital status, job category, and highest academic degree, and participant responses to the existing validated Arabic version of the NMP‐Q, which has excellent classical psychometric properties (Al‐Balhan et al. 2018).

### 2.4 Data Collection Procedures

Individuals received an invitation to participate in the study via instant messaging apps, email, and social media platform advertisements explaining the purpose, content, and procedure. Participation was voluntary and anonymous. The time required to complete data collection was ∼15 min. To broaden the sample, participants were requested to forward the online survey to family members and acquaintances. According to a recent meta‐analysis, this sampling approach allowed for data collection from a diverse community sample (Wang et al. 2020).

We employed several strategies to enhance the quality of the collected data. First, we used a CAPTCHA challenge to ensure a human was completing the survey and to avoid bots. The CAPTCHA was presented at the beginning of the survey, and its completion served as informed consent after reading the informed consent statement. Second, we used time cutoffs to exclude surveys that were completed in less than 100 s. This screened out the automatic, thoughtless response sets. Third, sequential duplicate responses (when someone mistakenly presses the send button more than once) were manually screened and eliminated by a research team member.

### 2.5 Ethical Considerations

Ethics approval was obtained from the institutional review board of the University of Jordan, Jordan. All participants were voluntary and did not need to send in their responses if they did not wish to. The act of completing and submitting the questionnaire was assumed to mean they understood the study content and consented to participating. All responses were anonymous and no remuneration or other incentives were provided.

### 2.6 Data Analysis

Descriptive statistics are reported for NMP‐Q items as follows: For CTT analysis, the arithmetic mean, standard deviation (SD), skewness, and kurtosis were reported. The arithmetic mean values describe the central tendency of responses for each item (Anjaria 2022). SD values quantify the dispersion of the responses (Anjaria 2022). Skewness and kurtosis characterize the shape of the response distributions (Seijas‐Macias et al. 2023). For IRT analysis, item difficulty and item discrimination parameters are reported. Difficulty parameters indicate the location of each item on the underlying trait continuum (Nguyen et al. 2014). Discrimination parameters indicate how well each item discriminates among participants at different levels of the underlying trait (Nguyen et al. 2014).

A polychoric correlation coefficient matrix of the NMP‐Q items was developed. Polychoric correlation coefficients provide information about the associations between each pair of NMP‐Q items, enabling an examination of the relationships among the items on the questionnaire (Olsson 1979). The polychoric correlation matrix is an essential component in evaluating the underlying factor structure of the NMP‐Q through techniques such as exploratory factor analysis (Olsson 1979).

The reliability of the NMP‐Q as a measure was extensively examined using several methods. The correlation coefficients of McDonald's *ω* (Zinbarg et al. 2005), Cronbach's *α* (Zinbarg et al. 2005), Guttman's *λ*2 (Oosterwijk et al. 2017), Guttman's *λ*6 (Oosterwijk et al. 2017), greatest lower bound (Malkewitz et al. 2023), average interitem correlation (Ferketich 1991), Feldt–Gilmer coefficient (Shu and Schwarz 2014), Feldt–Brennan coefficient (Shu and Schwarz 2014), and Raju's *β* were used (Warrens 2016). McDonald's *ω* and Cronbach's *α* evaluate reliability based on the interrelatedness of items (Zinbarg et al. 2005). Guttman's *λ*2 and *λ*6 are less biased estimates of interrelatedness compared to Cronbach's *α* (Oosterwijk et al. 2017). The greatest lower bound estimate indicates reliability based on common factors (Malkewitz et al. 2023). The average interitem correlation further examines the homogeneity of items. Feldt–Gilmer and Feldt–Brennan coefficients test reliability over multiple test forms (Ferketich 1991; Shu and Schwarz 2014). Raju's *β* statistic accounts for different sources of measurement error (Warrens 2016). For the average interitem correlation reliability, a value >0.40 indicates good reliability; for all other coefficients, a value >0.70 indicates good reliability.

Several fit indices were used to assess the fitness of the NMP‐Q, including the comparative fit index (CFI) (Xia and Yang 2019), Tucker–Lewis index (TLI) (Xia and Yang 2019), root mean square error of approximation (RMSEA) (Xia and Yang 2019), and standardized root mean square residual (SRMR) (Xia and Yang 2019). Reporting multiple fit indices enables a robust evaluation of how well the hypothesized four‐factor model reproduces the observed covariance matrix (Xia and Yang 2019). The fit index, CFI, and TLI compare the fitted model to a null model, with values close to 1.0 indicating good fit (Xia and Yang 2019). RMSEA and its confidence interval indicate a lack of fit per degree of freedom, with lower values indicating better fit. SRMR is the standardized difference between observed and predicted correlations, with values less than 0.08 indicating adequate fit (Xia and Yang 2019). For CCT, one‐ and four‐factor models were reported. For IRT, only the one‐factor model was used to allow item analysis of the entire questionnaire.

To select an abbreviated form of the NMP‐Q with superior psychometric properties, we first conducted a unifactor IRT to examine the unidimensionality assumption required for IRT. Thus, the graded response model (i.e., a type of polytomous IRT model) of the NMP‐Q was initially performed (De Ayala 1994). Specifically, the rating scale model was utilized given the polytomous nature of the NMP‐Q items. Item statistics from the rating scale model are reported, including the tau parameters, item measure, standard error (SE), infit, and outfit for each NMP‐Q item (Samejima 2011). The item measure provides information about item difficulty, while the SE reflects the precision of measurement. Infit and outfit assess how well each item fits the underlying measurement model. Additionally, threshold parameters (b1–b6) are reported, indicating the level of the trait at which a response in each category became more likely (Samejima 2011). The tau parameter refers to the discrimination parameter of an item (Samejima 2011).

Item discrimination parameters were then estimated using a graded response multidimensional IRT (MIRT) model with between‐item multidimensionality to account for the multidimensional factor structure revealed by previous analyses (Samejima 2011). This allowed the discrimination parameter for each item to be estimated separately for the general factor and each specific factor (Samejima 2011). Ten items were selected (based on MIRT loading) for the abbreviated NMP‐Q based on having the highest discrimination values on the general nomophobia factor while still providing adequate coverage of the four specific factors according to MIRT. This approach ensures the abbreviated form represents both the general construct and its multidimensional nature (Samejima 2011).

These provisional 10 items (50% of the original NMP‐Q) were summed to generate an NMP‐Q Short score. The NMP‐Q Short was interpreted as 50% of the original, so it has a maximum score of 70, and the severity of NMP increases with a higher score. A score of 10 or less indicates a lack of NMP, a score of 11–29 indicates mild NMP, a score of 30–49 indicates moderate NMP, and a score of 50 or more indicates severe NMP. To examine the performance of the NMP‐Q Short against the original NMP‐Q, correlation analysis was performed using the Pearson product‐moment correlation coefficient (*r*) for the raw scores and the intraclass correlation coefficient (ICC) for the severity categories.

R for statistical computing version 4.2.3 (Shortstop Beagle), released on March 15, 2023, was used to carry out statistical analyses and visualizations (R‐Core‐Team 2023). The packages “mirt” (Chalmers 2012) and “ggmirt” (Masur 2022) were used for all analytics and visualizations. A *p*‐value of <0.05 was considered statistically significant in all analyses.

## 3 Results

The mean NMP‐Q score for the total study sample was 78 (SD = 27, range = 20–140). Table 1 provides descriptive statistics for each item on the NMP‐Q using CCT and IRT analyses. For CCT analysis, an examination of the 20 items in the NMP‐Q reveals that the mean scores for the items range from 2.88 to 4.83, and the SDs range from 1.77 to 2.15. Negative skewness (−0.49 to −0.3) suggests that the item scores are slightly skewed to the left. Negative kurtosis (−0.6 to −1.31) indicates a relatively flatter distribution with lighter tails for all items.

**TABLE 1:** Descriptive results of Nomophobia Questionnaire (NMP‐Q).

	Classical test theory				Item response theory	
NMP‐Q item (#)	Mean	SD	Skewness	Kurtosis	Difficulty	Discrimination
1	4.83	1.77	−0.49	−0.60	3.15	0.37
2	4.60	1.85	−0.3	−0.94	3.09	0.38
3	3.30	1.90	0.44	−0.90	2.71	0.42
4	4.29	1.96	−0.14	−1.16	3.01	0.47
5	3.92	2.08	0.08	−1.29	2.73	0.42
6	3.93	2.12	0.05	−1.31	2.67	0.38
7	4.02	2.15	0.05	−1.39	2.59	0.41
8	3.33	2.09	0.41	−1.16	2.52	0.43
9	4.35	1.99	−0.17	−1.19	2.93	0.4
10	4.32	1.98	−0.16	−1.17	2.93	0.48
11	4.27	2.06	−0.15	−1.28	2.82	0.5
12	3.66	1.99	0.21	−1.14	2.81	0.59
13	4.03	1.92	0.02	−1.10	3.01	0.6
14	4.01	1.98	0.02	−1.19	2.96	0.52
15	3.93	1.93	0.04	−1.11	3.02	0.58
16	2.88	1.92	0.74	−0.62	2.36	0.55
17	3.54	1.98	0.26	−1.14	2.84	0.58
18	3.51	1.97	0.27	−1.12	2.79	0.59
19	3.32	1.93	0.37	−1.00	2.75	0.53
20	3.37	2.01	0.38	−1.07	2.62	0.51

For IRT analysis, the difficulty values indicate the level of the underlying trait being measured at which an item functions optimally. Higher values mean the item is more difficult to answer. Items #4, #13, #15, #2, and #1 appeared to be the most difficult items during testing. The discrimination values represent how well an item differentiates between respondents with abilities above and below the item's difficulty level. Higher values indicate better discrimination. Items #17, #15, #18, and #12 appeared to have better discrimination. High discrimination values suggest that those items strongly differentiated between participants with high and low NMP.

Table 2 provides a polychoric correlation coefficient matrix for the NMP‐Q items as ordinal variables. Correlation coefficients are all positive, with an average strength of 0.5. NMP‐Q Items #11 to #20 appear to cluster with stronger intercorrelations.

**TABLE 2:** Polychoric correlation coefficient matrix of Nomophobia Questionnaire (NMP‐Q).

NMP‐Q item (#)	#1	#2	#3	#4	#5	#6	#7	#8	#9	#10	#11	#12	#13	#14	#15	#16	#17	#18	#19	#20
1	1.00																			
2	0.67	1.00																		
3	0.45	0.59	1.00																	
4	0.53	0.67	0.64	1.00																
5	0.36	0.51	0.56	0.57	1.00															
6	0.33	0.41	0.47	0.49	0.57	1.00														
7	0.42	0.51	0.49	0.50	0.55	0.54	1.00													
8	0.32	0.40	0.47	0.42	0.45	0.50	0.51	1.00												
9	0.46	0.56	0.49	0.56	0.49	0.44	0.57	0.47	1.00											
10	0.38	0.49	0.48	0.50	0.51	0.43	0.48	0.52	0.58	1.00										
11	0.25	0.31	0.29	0.32	0.33	0.32	0.31	0.38	0.37	0.57	1.00									
12	0.27	0.38	0.45	0.40	0.42	0.35	0.40	0.38	0.42	0.51	0.61	1.00								
13	0.29	0.39	0.38	0.38	0.37	0.32	0.37	0.37	0.43	0.58	0.72	0.76	1.00							
14	0.25	0.33	0.33	0.31	0.35	0.30	0.35	0.30	0.38	0.46	0.60	0.68	0.73	1.00						
15	0.26	0.35	0.38	0.38	0.37	0.32	0.37	0.36	0.41	0.55	0.71	0.73	0.84	0.75	1.00					
16	0.21	0.35	0.43	0.35	0.36	0.33	0.38	0.39	0.41	0.37	0.53	0.67	0.60	0.54	0.62	1.00				
17	0.30	0.41	0.43	0.42	0.37	0.33	0.43	0.34	0.48	0.41	0.49	0.67	0.62	0.53	0.64	0.78	1.00			
18	0.27	0.40	0.43	0.39	0.35	0.34	0.43	0.35	0.45	0.44	0.50	0.69	0.66	0.60	0.67	0.75	0.82	1.00		
19	0.24	0.33	0.39	0.33	0.34	0.34	0.36	0.34	0.34	0.37	0.50	0.62	0.59	0.58	0.63	0.66	0.68	0.71	1.00	
20	0.28	0.37	0.39	0.40	0.39	0.38	0.42	0.38	0.43	0.39	0.52	0.65	0.59	0.52	0.60	0.68	0.73	0.73	0.63	1.00

Table 3 reports the results of various reliability coefficients for the NMP‐Q. According to CTT, the overall reliability estimates for the NMP‐Q ranged from 0.93 to 0.97, with 95% confidence intervals of [0.92, 0.94] to [0.95, 0.99]. These coefficients include McDonald's *ω*, Cronbach's *α*, Guttman's *λ*2, Guttman's *λ*6, the greatest lower bound, and average interitem correlations. When examining the deletion of individual NMP‐Q items, the reliability coefficients remain consistently high, ranging from 0.93 to 0.97. Removing any single item from the questionnaire did not significantly impact the overall reliability of the instrument.

**TABLE 3:** Reliability analysis of the Nomophobia Questionnaire (NMP‐Q).

	Classical test theory						Item response theory		
Correlation coefficient	McDonald's *ω*	Cronbach's *α*	Guttman's *λ*2	Guttman's *λ*6	Greatest lower bound	Average interitem	Feldt–Gilmer	Feldt–Brennan	Raju's *β*
Overall reliability [95% CI]	0.93 [0.92, 0.94]	0.94 [0.93, 0.95]	0.95 [0.94, 0.96]	0.97 [0.95, 0.99]	0.97 [0.95, 0.99]	0.42 [0.41, 0.43]	0.88 [0.87, 0.89]	0.88 [0.87, 0.89]	0.88 [0.87, 0.89]
Deletion of NMP‐Q 1	0.93	0.94	0.94	0.95	0.97	0.45	0.88	0.88	0.88
Deletion of NMP‐Q 2	0.93	0.93	0.94	0.95	0.96	0.60	0.88	0.88	0.88
Deletion of NMP‐Q 3	0.93	0.93	0.94	0.95	0.96	0.60	0.88	0.88	0.87
Deletion of NMP‐Q 4	0.93	0.93	0.94	0.95	0.96	0.61	0.87	0.87	0.87
Deletion of NMP‐Q 5	0.93	0.93	0.94	0.95	0.96	0.58	0.88	0.88	0.88
Deletion of NMP‐Q 6	0.93	0.94	0.94	0.95	0.97	0.53	0.88	0.88	0.88
Deletion of NMP‐Q 7	0.93	0.93	0.94	0.95	0.97	0.59	0.88	0.88	0.88
Deletion of NMP‐Q 8	0.93	0.94	0.94	0.95	0.97	0.53	0.88	0.88	0.87
Deletion of NMP‐Q 9	0.93	0.93	0.94	0.95	0.96	0.62	0.88	0.88	0.88
Deletion of NMP‐Q 10	0.93	0.93	0.94	0.95	0.96	0.64	0.87	0.87	0.87
Deletion of NMP‐Q 11	0.93	0.93	0.94	0.95	0.96	0.61	0.87	0.87	0.87
Deletion of NMP‐Q 12	0.93	0.93	0.93	0.95	0.96	0.73	0.87	0.87	0.87
Deletion of NMP‐Q 13	0.93	0.93	0.93	0.95	0.96	0.73	0.87	0.87	0.87
Deletion of NMP‐Q 14	0.93	0.93	0.94	0.95	0.96	0.64	0.87	0.87	0.87
Deletion of NMP‐Q 15	0.93	0.93	0.93	0.95	0.96	0.72	0.87	0.87	0.87
Deletion of NMP‐Q 16	0.93	0.93	0.93	0.95	0.96	0.66	0.87	0.87	0.87
Deletion of NMP‐Q 17	0.93	0.93	0.93	0.95	0.96	0.71	0.87	0.87	0.87
Deletion of NMP‐Q 18	0.93	0.93	0.93	0.95	0.96	0.72	0.87	0.87	0.87
Deletion of NMP‐Q 19	0.93	0.93	0.94	0.95	0.96	0.63	0.87	0.87	0.87
Deletion of NMP‐Q 20	0.93	0.93	0.93	0.95	0.96	0.68	0.87	0.87	0.87

IRT coefficients including Feldt–Gilmer, Feldt–Brennan, and Raju's *β* are also reported in Table 3. These coefficients estimate the reliability of the NMP‐Q based on the underlying item response model. The coefficients ranged from 0.87 to 0.88. No item was flagged for deletion to improve reliability coefficients.

The results of the CTT analysis fit indices for the NMP‐Q using different models and theories are presented in Table 4. For the four‐factor model, the CFI was 0.93, suggesting a good fit to the data. The TLI was 0.92, also indicating a good fit. The RMSEA was 0.07, suggesting a good fit to the data. The SRMR was 0.05, indicating a good fit. For the one‐factor model in CTT, the fit indices were lower compared to the four‐factor model; CFI was 0.72 and TLI was 0.70, indicating poorer fits. The RMSEA was 0.14 with a 90% CI of 0.12–0.15, suggesting a larger discrepancy from the observed data, and the SRMR was 0.10, indicating a higher level of residual error.

**TABLE 4:** Fit indices of the Nomophobia Questionnaire (NMP‐Q).

Fit index	Classical test theory (four factors)	Classical test theory (one factor)	Item response theory (one factor)	Item response theory (four factors)
Comparative fit index (CFI)	0.93	0.72	0.83	0.92
Tucker–Lewis index (TLI)	0.92	0.70	0.82	0.91
Root mean square error of approximation (RMSEA)	0.07	0.14	0.18	0.06
RMSEA 90% CI lower bound	0.06	0.12	0.17	0.05
RMSEA 90% CI upper bound	0.08	0.15	0.19	0.07
Standardized root mean square residual (SRMR)	0.05	0.10	0.11	0.055

In the analysis conducted with IRT using the one‐factor model, the CFI was 0.83 and the TLI was 0.82, both suggesting a relatively good fit. The RMSEA was 0.18 with a 90% CI of 0.17–0.19, indicating a moderate discrepancy between the model and the observed data. The SRMR was 0.11, suggesting a moderate level of residual error.

Table 5 provides tau parameters and item statistics for the polytomous‐graded IRT model of the NMP‐Q. The tau parameters provide information about the item's performance in the model, including its outfit and infit statistics. The item measures represent the estimated difficulty or severity of each item in relation to the construct being measured (nomophobia). Items with lower item measures (e.g., NMP‐Q Item #19 and NMP‐Q Item #20) indicate lower levels of nomophobia, while items with higher item measures (e.g., NMP‐Q Item #1 and NMP‐Q Item #2) suggest higher levels of nomophobia. The infit and outfit statistics measure the fit of each item to the rating scale model; items demonstrated good fit, as indicated by infit and outfit values around 1. Most items have discrimination parameters greater than 1, suggesting that they effectively differentiate between individuals with varying levels of nomophobia. The category threshold parameters (b1–b6) indicated that individuals with lower levels of nomophobia are more likely to choose higher response categories.

**TABLE 5:** Polytomous Graded Item Response Theory Model of the Nomophobia Questionnaire (NMP‐Q).

Item statistics of the rating scale model					Tau parameters^a^						
NMP‐Q item (#)	Item measure	SE	Infit	Outfit	a	b1	b2	b3	b4	b5	b6
1	−1.64	0.01	1.01	1.05	0.87	−3.52	−2.56	−1.72	−0.5	0.62	1.51
2	−1.53	0.01	1.00	1.01	1.24	−2.57	−1.67	−0.91	−0.12	0.61	1.31
3	−0.94	0.01	1.01	1.02	1.29	−1.22	−0.37	0.25	0.96	1.7	2.28
4	−1.38	0.01	1.02	1.06	1.29	−2.08	−1.24	−0.52	0.12	0.75	1.48
5	−1.22	0.01	1.01	1.01	1.19	−1.62	−0.84	−0.2	0.4	1.01	1.64
6	−1.22	0.01	1.01	1.02	1.04	−1.66	−0.91	−0.26	0.44	1.09	1.74
7	−1.26	0.01	1.01	1.03	1.26	−1.64	−0.75	−0.22	0.31	0.79	1.37
8	−0.95	0.01	1.02	1.03	1.12	−0.98	−0.27	0.26	0.89	1.52	2.17
9	−1.42	0.01	1.00	1.00	1.38	−2.01	−1.15	−0.55	0.04	0.66	1.3
10	−1.40	0.01	1.01	1.03	1.59	−1.79	−1.05	−0.51	0.11	0.64	1.22
11	−1.38	0.01	0.99	0.99	1.88	−1.49	−0.85	−0.37	0.09	0.57	1.1
12	−1.10	0.01	0.96	1.09	2.95	−0.98	−0.46	−0.04	0.41	0.91	1.31
13	−1.27	0.01	0.97	0.94	2.96	−1.31	−0.71	−0.23	0.24	0.75	1.18
14	−1.26	0.01	0.98	0.98	2.11	−1.38	−0.76	−0.21	0.25	0.77	1.31
15	−1.23	0.01	0.96	0.91	2.99	−1.19	−0.66	−0.21	0.3	0.78	1.25
16	−0.72	0.01	0.95	0.89	2.43	−0.46	0.02	0.4	0.87	1.36	1.74
17	−1.05	0.01	0.96	1.11	2.74	−0.91	−0.39	0.01	0.49	0.92	1.48
18	−1.03	0.01	0.95	1.19	2.92	−0.86	−0.38	0.04	0.45	0.99	1.43
19	−0.94	0.01	1.01	1.02	2.16	−0.8	−0.33	0.16	0.68	1.19	1.75
20	−0.96	0.01	0.98	1.00	2.34	−0.75	−0.29	0.13	0.62	1.11	1.52

*Note*: Infit, information‐weighted mean square statistic; Outfit, outlier‐sensitive mean square statistic.

^a^ Delta‐tau parameterization of the partial credit model.

Figure S1 shows the item probability traceline plots, and Figure S2 reports the item information traceline plot. Figure S3 shows item infit and outfit statistics, and Figure S4 shows person infit and outfit statistics.

Table 6 provides tau parameters and item statistics for the polytomous‐graded MIRT model of the NMP‐Q. From Table 6, 10 items were selected to create the NMP‐Q Short; these are Items #11, #13, #15, #17, #18, #20, #1, #2, #5, and #6. Selection of the items was based on loading, commonalities, tau parameters, and discrimination parameters. For example, NMP‐Q Item #11 has a high positive loading on F1 (0.782), suggesting a strong positive relationship between NMP‐Q Item #11 and F1. Higher commonality values indicate that a larger proportion of the item's variance is accounted for by the factors. For instance, NMP‐Q Item #13 has a high commonality (0.835), indicating that a significant portion of its variance is explained by the underlying factors. Negative tau parameter values indicate that the item is more challenging or severe. For example, NMP‐Q Item #1 has a negative tau parameter (−0.768), suggesting it is a challenging item. Higher discrimination parameters suggest better item discrimination. For example, NMP‐Q Item #13 has a high discrimination parameter (1.901), indicating that it effectively differentiates between individuals with varying levels of the underlying factors.

**TABLE 6:** Multidimensional item response theory (MIRT) model of the Nomophobia Questionnaire (NMP‐Q).

NMP‐Q item (#)	Factor loading				Commonality	Tau parameters^a^									
	F1	F2	F3	F4	h2	a1	a2	a3	a4	d1	d2	d3	d4	d5	d6
1	0.034	−0.053	**0.706**	0.053	0.539	−0.768	−0.633	−0.228	1.532	4.114	3.024	2.018	0.526	−0.868	−1.938
2	0.030	−0.011	**0.765**	0.123	0.737	−1.371	−1.007	−0.238	2.278	5.277	3.447	1.785	0.092	−1.436	−2.907
3	−0.061	0.088	0.331	0.433	0.516	−1.113	−0.852	0.248	1.03	1.858	0.498	−0.496	−1.651	−2.801	−3.677
4	−0.034	0.002	0.468	0.415	0.633	−1.223	−1.085	0.225	1.505	3.676	2.197	0.88	−0.312	−1.507	−2.842
5	−0.044	−0.019	0.013	**0.742**	0.518	−1.002	−1.024	0.751	0.708	2.381	1.202	0.22	−0.693	−1.601	−2.533
6	−0.086	0.010	−0.124	**0.788**	0.455	−0.876	−0.948	0.76	0.415	2.072	1.128	0.291	−0.615	−1.448	−2.265
7	−0.106	0.091	0.027	0.697	0.508	−1.114	−0.989	0.611	0.632	2.475	1.116	0.277	−0.555	−1.328	−2.218
8	0.027	0.000	−0.109	0.683	0.396	−0.832	−0.681	0.75	0.419	1.237	0.33	−0.353	−1.171	−1.977	−2.8
9	0.025	0.047	0.182	0.524	0.488	−1.083	−0.751	0.515	0.87	3.177	1.826	0.831	−0.16	−1.178	−2.206
10	0.399	−0.172	0.015	0.567	0.56	−1.064	−0.387	1.207	0.973	3.36	1.982	0.926	−0.251	−1.256	−2.362
11	**0.782**	−0.055	−0.049	0.094	0.609	−1.204	0.86	1.328	0.754	3.323	1.923	0.877	−0.174	−1.248	−2.412
12	0.547	0.319	−0.015	0.059	0.685	−2.017	0.856	1.044	0.631	2.989	1.409	0.128	−1.251	−2.81	−4.029
13	**0.863**	0.087	0.051	−0.050	0.835	−2.457	1.768	1.901	1.388	5.489	3.051	1.034	−0.908	−3.08	−4.871
14	0.732	0.108	0.027	−0.055	0.623	−1.432	1.042	1.062	0.73	3.317	1.84	0.531	−0.584	−1.825	−3.147
15	**0.840**	0.126	0.004	−0.043	0.826	−2.428	1.754	1.853	1.174	4.877	2.778	0.917	−1.171	−3.154	−5.041
16	0.121	0.693	−0.055	0.036	0.61	−2.048	0.488	0.312	0.04	1.159	−0.112	−1.095	−2.411	−3.727	−4.732
17	0.076	**0.740**	0.058	0.001	0.669	−2.361	0.44	0.159	0.227	2.759	1.046	−0.106	−1.54	−2.99	−4.608
18	0.121	**0.766**	−0.062	0.013	0.704	−2.521	0.647	0.337	0	2.819	1.102	−0.182	−1.518	−3.462	−4.831
19	0.069	0.720	0.026	−0.121	0.518	−1.684	0.53	0	0	1.741	0.653	−0.424	−1.623	−2.792	−4.005
20	−0.146	**0.755**	−0.002	0.132	0.54	−1.842	0	0	0	1.736	0.575	−0.375	−1.575	−2.815	−3.76

*Note*: F1–F4 represent the four factors of the NMP‐Q. F1, not being able to communicate; F2, loosing communication; F3, not being able to access the internet; F4, giving up convenience. Bold values represents “factor loading”.

^a^ Delta‐tau parameterization of the partial credit model.

The NMP‐Q Short yielded a score of 40 (SD = 13, range = 10–70). The correlation between the NMP‐Q Short and the NMP‐Q was 0.97 for both *r* and ICC.

## 4 Discussion

The purpose of this study was to use IRT methods to further advance our knowledge about the psychometric properties of the NMP‐Q. The results of our analytics provided strong psychometric support for the NMP‐Q as an NMP measure. The NMP‐Q exhibits a robust level of reliability supported by the high‐reliability coefficients of the nine different reliability metrics derived from the CTT analyses. Moreover, reporting the polychoric correlation matrix provided transparency regarding the correlations among the NMP‐Q items, which indicated a high level of consistency and that the items of the questionnaire are measuring a similar construct. Furthermore, no item was suggested for removal to improve reliability indices. These results are consistent with those of previous studies (Jahrami et al. 2023). Moreover, the fit indices from the different models and theories suggest that the four‐factor model in CTT provided the best fit to the data. Subsequently, the one‐factor models in both CTT and IRT analyses demonstrated comparatively weaker fits to the data, indicating that while they may provide simplified representations of the questionnaire, they do not capture the multidimensional nature of NMP as effectively as the four‐factor model. The better fit of the four‐factor model demonstrates that taking into account the multiple dimensions of NMP should be standard when conducting future assessments and interpreting findings (Jahrami et al. 2023).

The four‐factor model showed superior fit, but the unidimensional IRT approach for the NMP‐Q Short was chosen for clinical and research utility. The short version aims to provide a rapid screening tool that captures the overall severity of nomophobia rather than its specific dimensions. IRT's unidimensional framework optimizes item selection to discriminate across a broad spectrum of general nomophobia severity, ensuring strong measurement precision for global assessment. The selected items—modeled unidimensionally—were derived from a multidimensional IRT analysis, reflecting the four underlying factors (e.g., Items #11 and #13 loaded strongly on Factor 1, while #17 and #18 aligned with Factor 2). This approach balances brevity with construct representation, as shown by the high correlation (*r* = 0.97) between the short and full versions. The NMP‐Q Short is suitable for quick screening, while the full NMP‐Q is recommended for nuanced, dimension‐specific analyses.

The RMSEA of 0.18 of the IRT one‐factor model indicates a suboptimal fit, highlighting the challenges associated with enforcing strict unidimensionality on a construct with established multidimensionality, as evidenced by the superior fit of the four‐factor CTT model (CFI = 0.93, RMSEA = 0.07). This misfit likely results from the attempt to consolidate items from distinct nomophobia domains (e.g., communication anxiety and loss of connectedness) into a single latent trait, which contravenes the IRT assumption of local independence. Nevertheless, the unidimensional approach was intentionally maintained for the NMP‐Q Short to enhance clinical practicality, allowing for a swift global assessment of nomophobia severity.

The tau parameters and item statistics suggest that the items in the NMP‐Q demonstrate a good fit to the polytomous graded IRT model, with appropriate item difficulty and precision. The “NMP‐Q Short,” consisting of 10 more informative items derived from the IRT analysis, maintains a strong correlation (i.e., *r* = 0.97) with the original NMP‐Q. This development illustrates the iterative process of questionnaire improvement, ensuring that only the most informative and sound psychometric items are used to assess NMP. The NMP‐Q Short could be a valuable tool for researchers and clinicians seeking a concise assessment of NMP, particularly for rapid screening or testing purposes. Nevertheless, practitioners aiming to explore specific facets or dimensions of NMP in‐depth would best continue to use the full NMP‐Q to obtain a more comprehensive assessment.

The items selected for the NMP‐Q Short were prioritized not only for their strong psychometric properties (e.g., high discrimination parameters) but also for their clinical relevance in capturing the core facets of nomophobia. To illustrate, items such as “I would be anxious because I could not keep in touch with my family and/or friends” (Item #13) and “I would feel anxious because my constant connection to my family and friends would be broken” (Item #15) directly reflect the DSM‐5‐aligned anxiety components of nomophobia, including the fear of disconnection and loss of control. These items correspond to real‐world functional impairments, such as compulsive checking behaviors or distress during device separation, which are critical targets for clinical intervention. Additionally, items like “I would be uncomfortable because I could not stay up‐to‐date with social media and online networks” (Item #17) address the social dependency dimension, a hallmark of severe nomophobia associated with interpersonal dysfunction. By retaining items that encompass communication, information access, and emotional dependency domains, the NMP‐Q Short ensures clinical relevance while maintaining brevity, enabling practitioners to identify high‐risk individuals and tailor interventions to specific behavioral or cognitive vulnerabilities. This balance highlights its utility as both a screening tool and a foundation for targeted therapeutic strategies.

## 5 Strengths and Limitations

This research has several strengths. First, the large sample size (*N* = 5087) provided good statistical power for detecting differences in questionnaire item functioning. This allowed for more precise item parameter estimates. Second, using IRT provided an evaluation of the measurement properties of the NMP scale and showed how well each item contributed to the overall construct being measured. Furthermore, IRT methods identified potentially problematic items that were not functioning well or not contributing useful information.

This study also has several limitations. First, examining only an Arabic version of the NMP‐Q means that our findings cannot be generalized to individuals experiencing NMP in other linguistic and cultural settings. While the original NMP‐Q has shown cross‐language validity, the abbreviated version needs thorough cultural adaptation and validation. Future studies should evaluate the NMP‐Q Short's psychometric properties across diverse populations, addressing variations in smartphone dependency norms and anxiety expression. Culturally informed translation and confirmatory analyses are essential for global applicability. Second, the use of IRT assumes unidimensionality, leading to the development of NMP‐Q Short, which does not report scorable known dimensions of NMP. Third, complementary validity evidence (e.g., convergent validity and discriminant validity) to better understand how NMP‐Q Short performs in our population is needed. Fourth, it is still unknown if the NMP‐Q Short is sensitive to changes in NMP over time or in response to interventions. Understanding how well NMP‐Q Short captures changes in NMP severity and whether it is responsive to therapeutic interventions is essential to its ultimate usefulness. Fifth, the convenience and snowball sampling strategies may introduce selection bias, potentially overrepresenting individuals with higher levels of smartphone engagement or social media connectivity. This limitation affects the generalizability of the findings to broader populations, particularly those with diverse technological access or demographic characteristics. It may also be beneficial that future cutoff scores (mild/moderate/severe) based on receiver operating characteristic (ROC) analysis are necessary, although this is not mandatory. Furthermore, establishing cutoff scores for mild, moderate, and severe NMP levels using ROC analysis for the short version could be valuable for future research.

## 6 Conclusions

The application of the IRT methodology provided robust evidence for the reliability and validity of the NMP‐Q for assessing individual differences in NMP. The measure provides an efficient, precise, and useful self‐report tool for further research on NMP as a psychological construct. This study helps support the ongoing development and optimization of the NMP‐Q as a psychometric instrument and highlights the fact that certain items appear to be of most importance. The NMP‐Q Short, containing only these best‐performing items, correlates very well with the full NMP‐Q, suggesting that, for rapid screening or testing, the NMP‐Q Short provides a viable option. Nevertheless, the NMP‐Q Short is still in its early stages of development, and future studies, particularly those involving complementary validity assessments, are needed.

## Author Contributions

**Haitham Jahrami**: conceptualization, investigation, writing – original draft, methodology, validation, visualization, writing – review and editing, software, formal analysis, project administration, data curation, supervision, resources. **Khaled Trabelsi**: conceptualization, investigation, writing – original draft, methodology, validation, visualization, writing – review and editing, software. **Achraf Ammar**: conceptualization, investigation, writing – original draft, methodology, validation, visualization, writing – review and editing, software, data curation, supervision, resources, project administration. **Nicola Luigi Bragazzi**: conceptualization, writing – original draft, writing – review and editing. **Hadeel Ghazzawi**: conceptualization, writing – original draft, writing – review and editing. **Waqar Husain**: writing – original draft, writing – review and editing, conceptualization. **Maha M. AlRasheed**: conceptualization, writing – original draft, writing – review and editing, data curation. **Seithikurippu R. Pandi‐Perumal**: writing – original draft, conceptualization, writing – review and editing. **Amir Pakpour**: conceptualization, writing – original draft, writing – review and editing. **Zahra Saif**: conceptualization, writing – original draft, writing – review and editing. **Michael V. Vitiello**: conceptualization, writing – original draft, writing – review and editing.

## Ethics Statement

The research received approval from the Institutional Review Board at the University of Jordan, Jordan (REC/Q1/02/2021R3). All methods were conducted in accordance with relevant guidelines and regulations. The study procedures adhered to the ethical guidelines outlined in the Helsinki Declaration of 1964 and its later amendments (1975, 1983, 1989, and 1996).

## Consent

Informed consent was obtained from all subjects.

## Conflicts of Interest

The authors declare no conflicts of interest.

## Peer Review

The peer review history for this article is available at https://publons.com/publon/10.1002/brb3.70622.

## Supporting information




**Supporting Figure 1**: Item probability traceline plots.

**Supporting Figure 2**: Item information traceline plots.

**Supporting Figure 3**: Item infit and outfit statistics.

**Supporting Figure 4**: Person infit and outfit statistics.

## Data Availability

The data that support the findings of this study are available from the corresponding author upon request.
