# Supplementary figures and images for: The Validation of the Nomophobia Questionnaire Using a Modern Psychometric Approach: An Item Response Theory Analysis of 5087 Participants
Source: Brain Behav. 2025 Jun 10;15(6):e70622. doi: 10.1002/brb3.70622 (PMC12152268; doi:10.1002/brb3.70622)

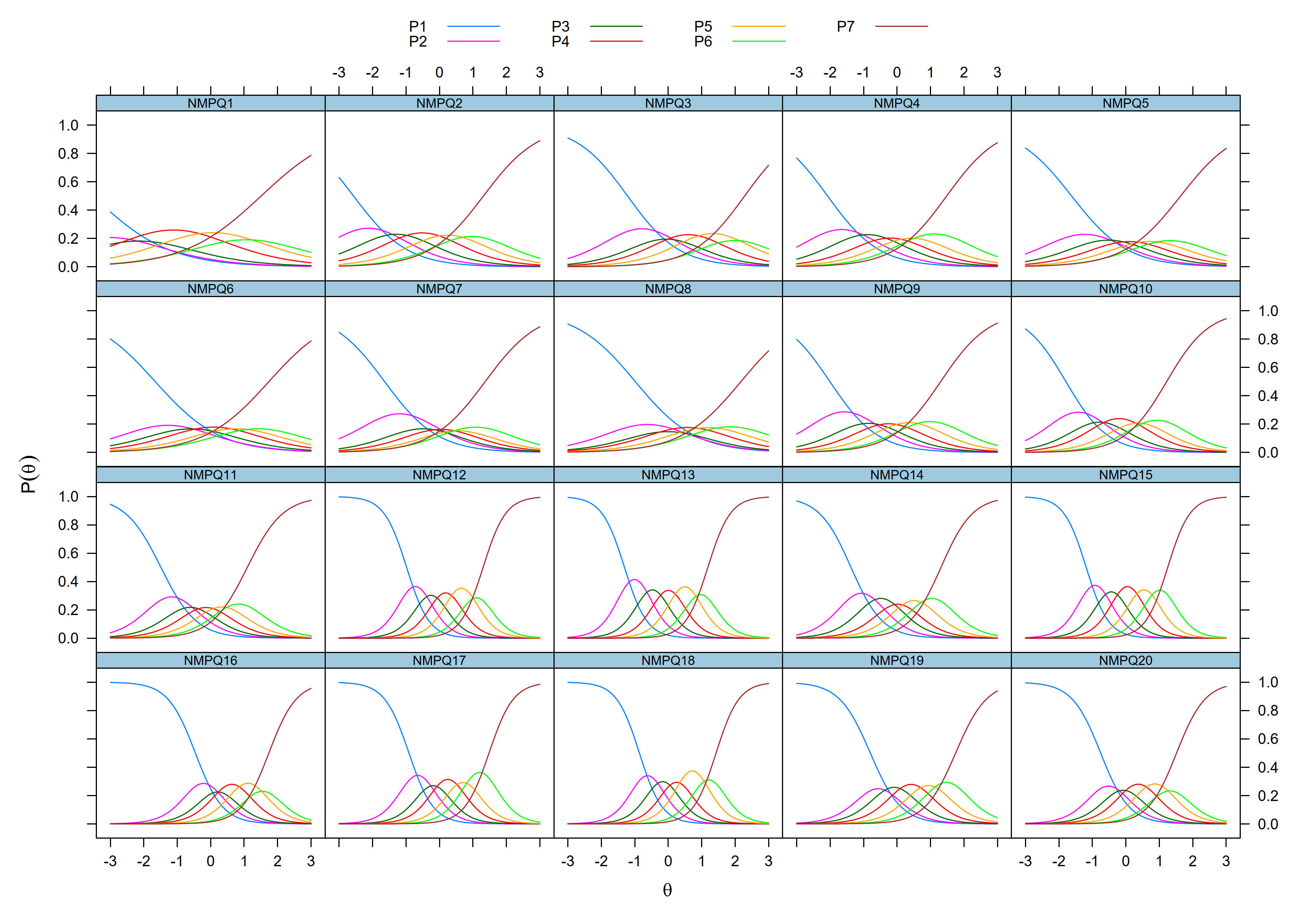

Supplement: Supplementary file 1 — Supporting Figure 1: Item probability traceline plots. [file BRB3-15-e70622-s001.jpg]

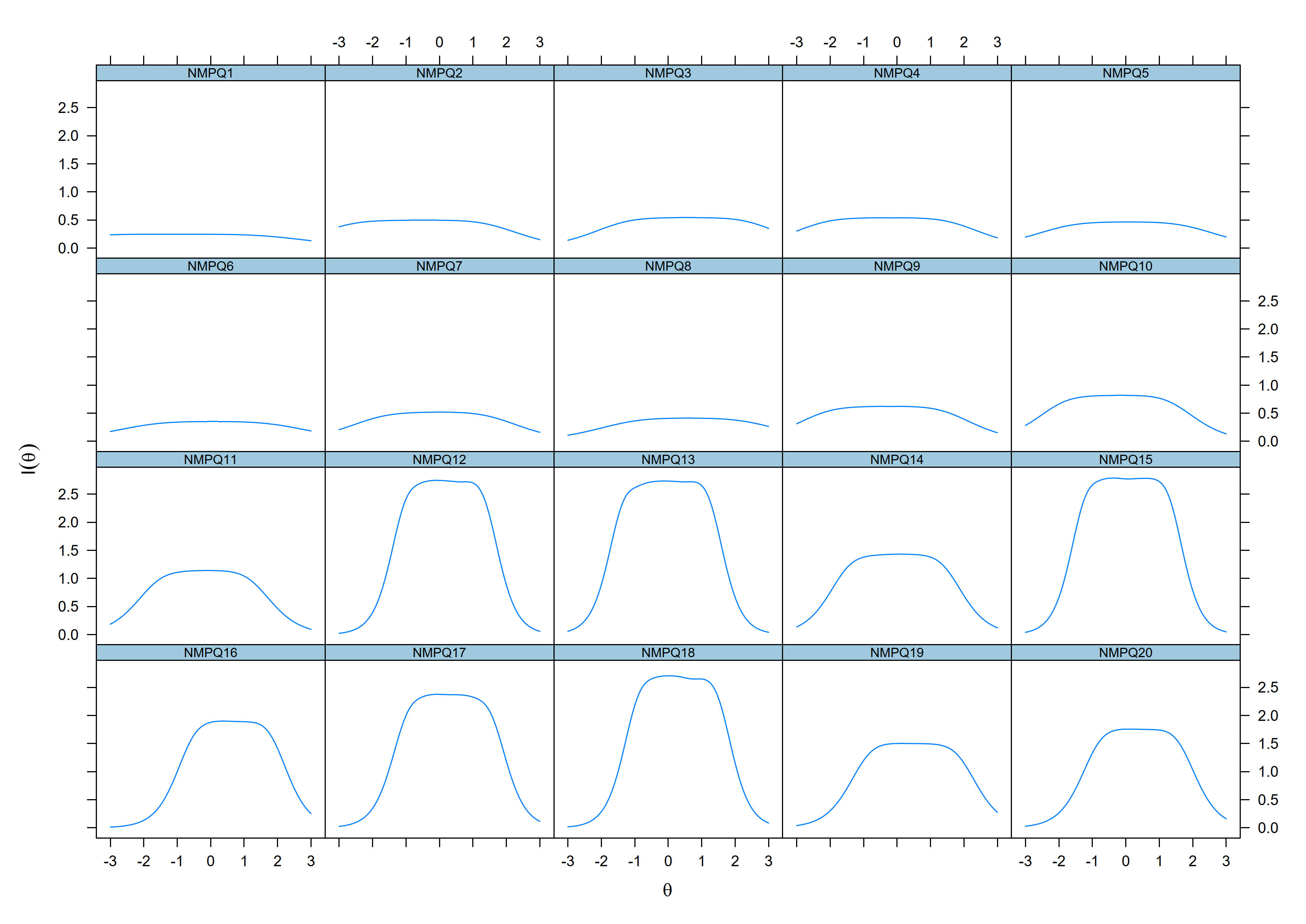

Supplement: Supplementary file 2 — Supporting Figure 2: Item information traceline plots. [file BRB3-15-e70622-s004.jpg]

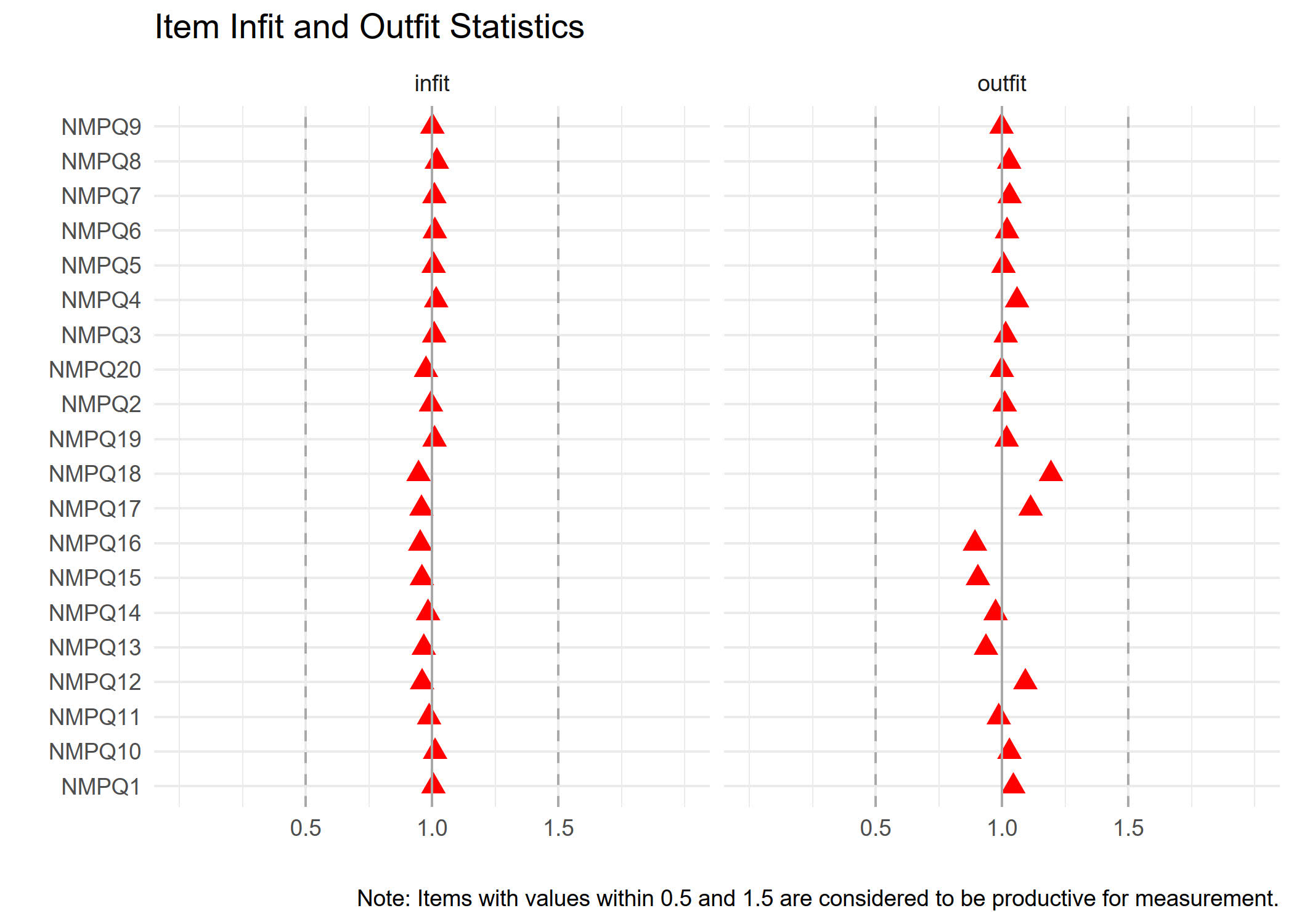

Supplement: Supplementary file 3 — Supporting Figure 3: Item infit and outfit statistics. [file BRB3-15-e70622-s002.jpg]

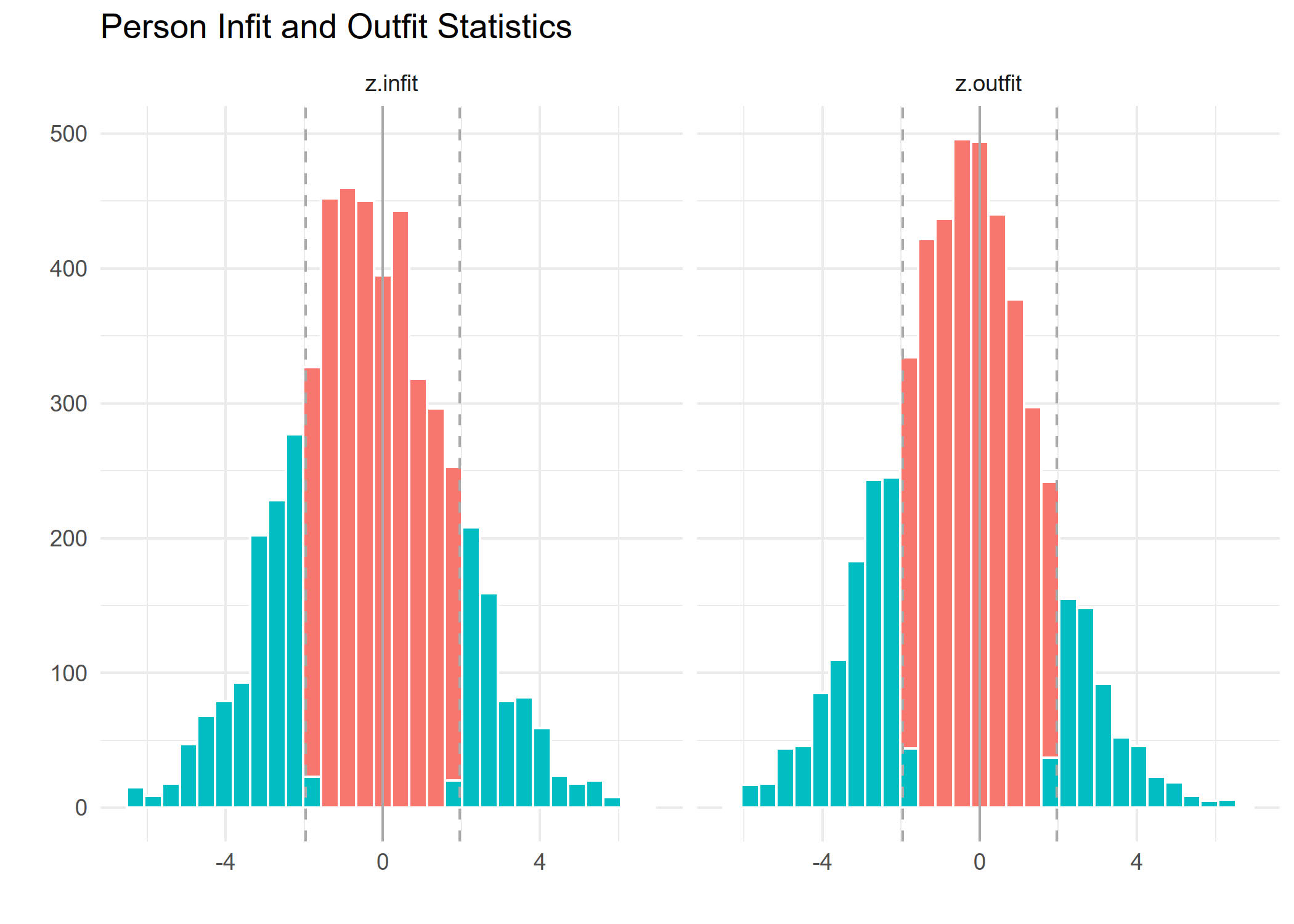

Supplement: Supplementary file 4 — Supporting Figure 4: Person infit and outfit statistics. [file BRB3-15-e70622-s003.jpg]
